# Involvement of the cervical cord and medulla in posterior reversible encephalopathy syndrome

**DOI:** 10.4103/0256-4947.75790

**Published:** 2011

**Authors:** Naseer A. Choh, Majid Jehangir, Muddassir Rasheed, Tajamul Mira, Irfan Ahmad, Suhil Choh

**Affiliations:** a Department of Radiodiagnosis, Shri Maharaja Hari Singh Hospital, Srinagar, India; b Department of Internal Medicine, Shri Maharaja Hari Singh Hospital, Srinagar, India; ^c^Department of Pediatrics, Shri Maharaja Hari Singh Hospital, Srinagar, India

## Abstract

The posterior reversible encephalopathy syndrome (PRES) is characterized by patchy cortical and subcortical lesions in the distribution of the posterior circulation. The lesions are classically reversible. This syndrome has multiple etiologies, most of which cause acute hypertension. We present a case of PRES with involvement of the medulla and cervical cord (apart from the typical parieto-occipital lesions)-an extremely rare imaging manifestation of PRES. It is important to recognize the imaging findings of PRES in spinal cord, and avoid misdiagnosis as myelitis by proper clinical correlation. Typically patients with myelitis have a profound neurodeficit, while patients with spinal manifestations of PRES are asymptomatic. Involvement of the cord in PRES has probably been an underrecognized entity as spinal imaging is not routinely performed in posterior reversible encephalopathy syndrome.

Posterior reversible encephalopathy syndrome (PRES) is a neurotoxic state accompanied by unique imaging findings. It is associated with a number of clinical conditions like preeclampsia, eclampsia, autoimmune diseases (e.g., systemic lupus erthematosus), allogeneic bone marrow and solid organ transplantation, shock and sepsis. Clinically, PRES is characterized by headache, vomiting, seizures, altered consciousness, and blindness.^1^ Imaging findings include symmetrical hyperintense lesions on T2 images in the parieto-occipital regions in typical cases. Holo-hemispheric, superior frontal sulcus, and uncommonly, involvement of brainstem, pons, cerebellum, basal ganglia, and splenium have also been described.^2^,^3^ The involvement of the medulla and cervical cord is very rare, with only few cases reported.^4^,^5^ We report a case of PRES with involvement of the cervical cord and medulla, in addition to typical parieto-occipital lesions.

## CASE

**Figure 1:**
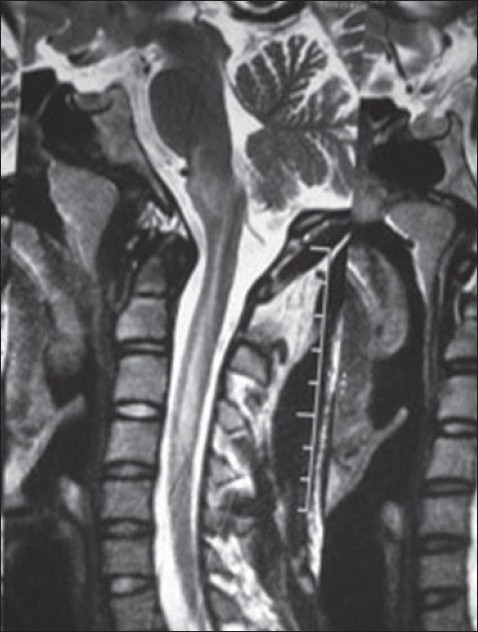
T2 wt sagittal image of cervical spine showing a hyperintense signal of the medulla and cervical cord, a rare manifestation of PRES.

**Figure 2:**
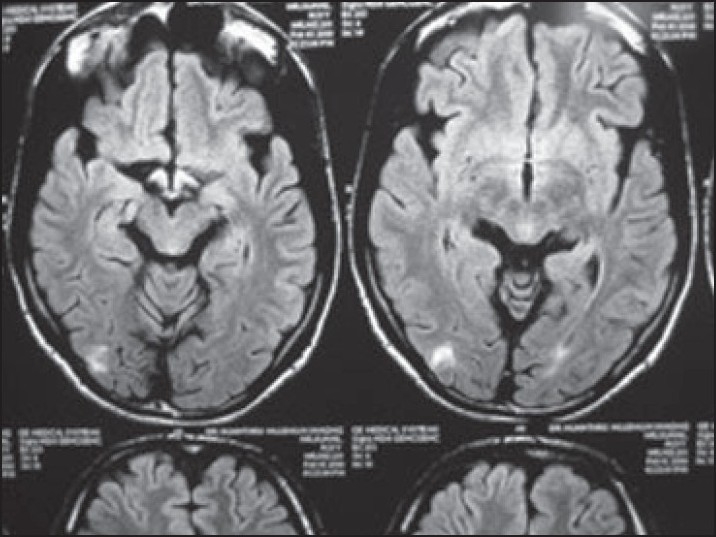
FLAIR image showing a hyperintense signal in the parietooccipital regions; these lesions are typical of PRES.

**Figure 3:**
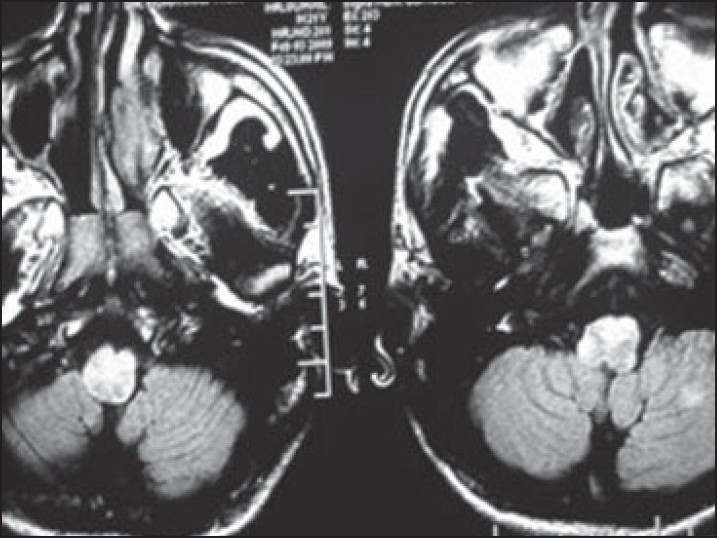
The medullary region showing a hyperintense signal on FLAIR, an atypical and rare distribution of PRES.

A 17-year-old male presented with a few days history of severe headache, visual disturbances, and a few episodes of vomiting. There was no history of fever, altered consciousness, seizures, or upper or lower limb weakness. The past medical history was unremarkable. At the time of examination, his BP was 240/130 mm Hg. The fundus examination revealed bilateral papilledema with grade 4 hypertensive changes. The neurological examination revealed normal grade 5 power in all four limbs with normally elicited deep tendon reflexes. The sensory examination was unremarkable with normal flexor plantar response bilaterally. Serum electrolytes were normal; kidney function tests were deranged (blood urea 86 mg/dL, serum creatinine 3.7 mg/dL). A renal biopsy revealed features of IgA nephropathy. MRI of the brain revealed a hyperintense signal of medulla and cervical cord on T2 and FLAIR images (**Figures 1–3**); small hyperintense foci were also noted in the parieto-occipital regions and deep cerebellar white matter. The lesions were also hyperintense on diffusion-weighted imaging (because of T2 shine-through); there was no evidence of restricted diffusion on apparent diffusion co-efficient maps. As there was no clinical or lab evidence to support the diagnosis of encephalomyelitis or acute disseminating encephalomyelitis (ADEM) as suggested initially by the radiologist, an atypical variant of PRES was considered as the most plausible diagnostic possibility. Further investigations including CSF studies were not considered necessary in view of the strongly suggestive clinical and imaging features. The patient rapidly improved with antihypertensive treatment and a repeat MRI done 1 month afterward showed resolution of the earlier imaging findings.

## DISCUSSION

PRES typically involves the parieto-occipital lobes (cortical/subcortical white matter). The atypical patterns of involvement include superior frontal sulcus pattern, temporal lobes (40%), cerebellar white matter (30%), basal ganglia (14%), and brainstem (13%); the splenium was involved in 10% of cases.^2^,^3^,^6^ Recurrent episodes of PRES may also occur, but are rare. Unilateral involvement and hemorrhage in the lesions is seen in 5% of patients, and permanent changes in the form of small lesions occurred in 26% in one large series.^7^ Clinical features include seizures (87%), encephalopathy (92%), visual symptoms (39%), and headache (53%).^7^

The most accepted explanation for the imaging finding in PRES is the autoregulatory failure in severe hypertension in the posterior circulation (because of poor sympathetic neural supply in the same); this results in dilitation of constricted arterioles, resulting in increased perfusion pressure, disruption of the blood-brain barrier and vasogenic edema. However, in 25% of patients with PRES, hypertension is absent or mild, and autoregulatory failure cannot explain the findings.^8^ The alternative theory holds that there is endothelial dysfunction (e.g., in sepsis, organ transplantation, pre-eclampsia) with subsequent vasoconstriction or leukocyte trafficking or both. This leads to vasculopathy and hypoperfusion, explaining the watershed appearance of PRES lesions on CT/MR imaging. According to this theory, autoregulatory vasoconstriction superimposed on toxicity vasoconstriction/hypoperfusion with borderzone ischemia could be responsible for beneficial effect of antihypertensive/magnesium management.^8^,^9^

There have been few case reports of involvement of the brainstem in PRES.^10^,^11^ The involvement of medulla oblongata and especially the cervical cord, however, is very rare.^4^ The imaging differential in such patients is brainstem infarction or ADEM, which can be distinguished by clinical grounds from PRES. Patients with ADEM have a history of fever, preceding viral illness, and a more profound neurodeficit. Massive brainstem infarction has a sudden onset and patients are comatose at presentation.^4^ The involvement of the cervical cord is exceedingly rare, and to our knowledge only two cases have been reported in the literature; one of the patients had cocaine-induced malignant hypertension.^5^,^12^ Thus, our patient had a very rare neuroimaging manifestation of PRES, namely the involvement of cervical cord as well as medulla, and was initially reported as ADEM; this was not consistent with the clinical features (the patient had no history of fever/motor or sensory deficit to suggest myelopathy, either historically or on examination).

The hyperintense signal seen on diffusion weighted images was likely due to the T2 shine-through phenomenon; this is not a rare finding in PRES and may be seen in as many as 28% patients.^3^ Diffusion restriction on ADC maps, or low or pseudonormalized ADC values suggest cytotoxic edema and potentially irreversible infarction; this was seen in 17% of patients in one series and portends a poor prognosis.^3^ There was no evidence of diffusion restriction in our case on the ADC map consistent with vasogenic edema. In view of the clinical features (severe hypertension, papilledema and evidence of renal parenchymal disease with recent onset of visual disturbances) and typical imaging features (bilateral parieto-occipital hyperintense lesions with involvement of medulla), the diagnosis of atypical PRES was kept as a first diagnostic possibility and no further lab investigations, including CSF analysis was done. The patient had dramatic improvement, and was discharged 1 week later. It is also pertinent to mention that apart from the cervicomedullary involvement, which is a rare phenomenon in PRES, our patient also had the typical parieto-occipital lesions that gave the initial clue to the diagnosis. The follow-up MRI, done 1 month after the initial imaging, showed complete resolution of the signal abnormality.

The cord involvement in this patient is likely to have same pathophysiology as the other classical neuroparenchymal lesions (autoregulatory failure with resultant vasogenic edema); however, other mechanisms may also be operative considering the rarity of cord involvement in PRES patients. We are of the opinion that the spinal form of PRES is probably underdiagnosed (as cord imaging is usually not done in PRES patients) or misdiagnosed as myelitis, and a correct diagnosis requires a high index of suspicion in the appropriate clinical setting. It also needs to be seen whether any particular etiology predisposes to cord involvement in PRES, as well as the therapeutic and prognostic implications that might entail.
